# Aging, cancer, and autophagy: connections and therapeutic perspectives

**DOI:** 10.3389/fmolb.2024.1516789

**Published:** 2025-01-28

**Authors:** Begoña Zapatería, Esperanza Arias

**Affiliations:** ^1^ Department of Medicine (Marion Bessin Liver Research Center), Department of Pathology, Albert Einstein College of Medicine, Bronx, NY, United States; ^2^ Einstein Aging Research Center, Montefiore Einstein Comprehensive Cancer Center, Albert Einstein College of Medicine, Bronx, NY, United States

**Keywords:** aging, cancer, autopaghy, proteostasis, therapeutics

## Abstract

Aging and cancer are intricately linked through shared molecular processes that influence both the onset of malignancy and the progression of age-related decline. As organisms age, cellular stress, genomic instability, and an accumulation of senescent cells create a pro-inflammatory environment conducive to cancer development. Autophagy, a cellular process responsible for degrading and recycling damaged components, plays a pivotal role in this relationship. While autophagy acts as a tumor-suppressive mechanism by preventing the accumulation of damaged organelles and proteins, cancer cells often exploit it to survive under conditions of metabolic stress and treatment resistance. The interplay between aging, cancer, and autophagy reveals key insights into tumorigenesis, cellular senescence, and proteostasis dysfunction. This review explores the molecular connections between these processes, emphasizing the potential for autophagy-targeted therapies as strategies that could be further explored in both aging and cancer treatment. Understanding the dual roles of autophagy in suppressing and promoting cancer offers promising avenues for therapeutic interventions aimed at improving outcomes for elderly cancer patients while addressing age-related deterioration.

## 1 Introduction

Aging is a progressive, multifaceted process characterized by evolving cellular and molecular deterioration that leads to a decline in physiological function and an increase in susceptibility to diseases, including cancer.

The intersection of aging and oncology has gained significant attention as cancer is predominantly an aging-associated disease especially in individuals over 65 years. The molecular mechanisms of aging are closely linked to tumorigenesis, influencing both age-related decline and cancer development.

Understanding the connections between aging and cancer, opens new avenues for therapeutic interventions. Recent advances in molecular biology show how aging-related changes in the microenvironment, immune surveillance, or systemic inflammation, promote cancer. In addition, cancer and its treatments can accelerate aging, shortening health and lifespan, also in cancer survivors.

This review explores the intricate relationship between aging and cancer -with a focus on autophagy, highlighting key molecular insights and therapeutic advances that hold promise for improving outcomes in elderly and older cancer patients.

## 2 Mechanisms of aging

Aging is characterized by the gradual loss of homeostasis due to the decline physiological functions and biological integrity (Aunan et al., 2016; Hunt et al., 2019). It involves changes at molecular, cellular, organ, and body levels. Extensive research has identified key drivers of aging, initially defined by nine hallmarks (López-Otín et al., 2013) and recently expanded to twelve (López-Otín et al., 2023). These **hallmarks of aging** are categorized into: primary, antagonistic, and integrative (López-Otín et al., 2013).

**Primary** hallmarks include genomic instability, which refers to accumulation of DNA damage causing cellular dysfunction. Telomere attrition, the shortening of telomeres during cell division, leads to cellular senescence or apoptosis (López-Otín et al., 2023). Epigenetic alterations, such as changes in DNA methylation and chromatin remodeling, contribute to age-related diseases (López-Otín et al., 2023). The loss of proteostasis is another crucial hallmark, linked to age-related disorders through the accumulation of damaged and misfolded proteins (Tsakiri et al., 2013; Gordon et al., 2014; Bobkova et al., 2015; Munkácsy et al., 2019; Dong et al., 2021). Autophagy, essential for maintaining proteostasis, is a significant hallmark of aging. Studies shows that impaired protein homeostasis and reduced autophagy accelerate aging. Conversely, interventions that enhance proteostasis and boost autophagic activity can slow the aging process (Rangel et al., 2022). Studies highlight that promoting protein quality control and autophagic flux helps mitigate the progression of aging and its associated pathologies (Pyo et al., 2013; Fernández et al., 2018; Wang et al., 2022).

**Antagonistic** hallmarks of aging include deregulated nutrient-sensing pathways, such as insulin/IGF-1 signaling, PI3K-AKT, mTOR, AMPK, and Ras-MEK-ERK. While these pathways promote growth during youth, their prolonged activation contributes to aging by promoting excessive anabolism, inflammation, and inhibiting autophagy (López-Otín et al., 2023). Reduced pathway activity has been linked to extended lifespan and improved health span (López-Otín et al., 2013). Mitochondrial dysfunction, marked by declining bioenergetics and increased reactive oxygen species (ROS), contributes to inflammation and cell death as aging progresses (Amorim et al., 2022). Cellular senescence triggered by telomere shortening, or other stressors, results in stable cell cycle arrest (Campisi and d'Adda di Fagagna, 2007; Collado et al., 2007; Kuilman et al., 2010). Although senescence reduces oncogenesis risks, the accumulation of senescent cells with age impairs tissue function (Tuttle et al., 2020).

**Integrative** hallmarks include stem cell exhaustion, altered intercellular communication, chronic inflammation, and dysbiosis. Stem cell exhaustion leads to reduced tissue regeneration due to depletion and functional decline accelerating aging (López-Otín et al., 2023). Altered intercellular communication disrupts homeostasis and stress response, promoting chronic inflammation and impaired immune surveillance (López-Otín et al., 2023). Chronic inflammation, or “*inflammaging*” is linked to arteriosclerosis, neuroinflammation, osteoarthritis, and intervertebral disc degeneration (López-Otín et al., 2023). Aging is associated with an increase in circulating proinflammatory cytokines, which promote inflammation as immune function declines (Hirata et al., 2020; Mogilenko et al., 2021). Research suggests that modulating immune pathways and using anti-inflammatory treatments may improve health span (D'Souza et al., 2021; Sciorati et al., 2020; Marín-Aguilar et al., 2020). Dysbiosis, an imbalance in the gut microbiome, impairs communication with the nervous system and organs (López-Otín and Kroemer, 2021), contributing to diseases as obesity, type 2 diabetes, and cancer (Zmora et al., 2019).

## 3 Cancer development

Cancer involves diseases characterized by uncontrolled cell growth, which can invade nearby tissues and metastasize. It affects almost any tissue, presenting as solid tumors or blood malignancies.

Cancer, **similarly to aging**, is defined by key hallmarks. Initially, six hallmarks were identified, later expanded to eight: self-sufficiency in growth signals, insensitivity to anti-growth signals, evasion of apoptosis, limitless replicative potential, sustained angiogenesis, tissue invasion and metastasis, reprogrammed energy metabolism, and immune evasion (Hanahan, 2022). Cancer transformation mechanisms differ due to specific mutations.

Cancer cells differ from normal cells in their autonomous growth. They achieve this through autocrine signaling, receptor overexpression, and altered pathways. Additionally, cancer cells modify their microenvironment to support growth (Hanahan, 2022; Hanahan and Weinberg, 2000).

Cancer cells resistance to antiproliferative signaling occurs through mutations in pro-apoptotic genes like p53 and activation of survival pathways such as PI3K-AKT/PKB (Hanahan, 2022; Hanahan and Weinberg, 2000). Their limitless replicative potential allows continuous division (Hanahan, 2022; Hanahan and Weinberg, 2011), often supported by upregulated telomerase or alternative telomere maintenance mechanisms (Blasco, 2005; Shay and Wright, 2000). Angiogenesis is vital for tumor growth, as it supplies nutrients and oxygen. Tumors achieve this by tipping the balance toward pro-angiogenic factors, like VEGF, over inhibitors (Hanahan, 2022; Hanahan and Weinberg, 2000).

Altered energy metabolism is another feature. Many cancer cells shift from oxidative phosphorylation to glycolysis (the Warburg effect) (Hanahan, 2022; Hanahan and Weinberg, 2011), boosting glucose uptake for biosynthesis and growth. Hypoxic conditions in tumors further promote glycolysis, where hypoxic cells generate lactate used by others through the TCA cycle (Semenza, 2008; Feron, 2009; Kennedy and Dewhirst, 2010). This shift, driven by oncogenes such as RAS, MYC, and TP53, is a hallmark of cancer (DeBerardinis et al., 2008; Jones and Thompson, 2009). Proteostasis and autophagy manage cancer cells’ metabolic demands, and their dysregulation is linked to tumor progression and treatment resistance, positioning autophagy as a potential therapeutic target.

The role of immune evasion as a core hallmark is debated, though evidence from human cancers and studies in immunodeficient mice indicates the immune system’s role in tumor control (Hanahan, 2022; Hanahan and Weinberg, 2011). Cancer’s hallmarks are facilitated by genomic instability and tumor-promoting inflammation (Teng et al., 2008; Kim et al., 2007).

**FIGURE 1:**
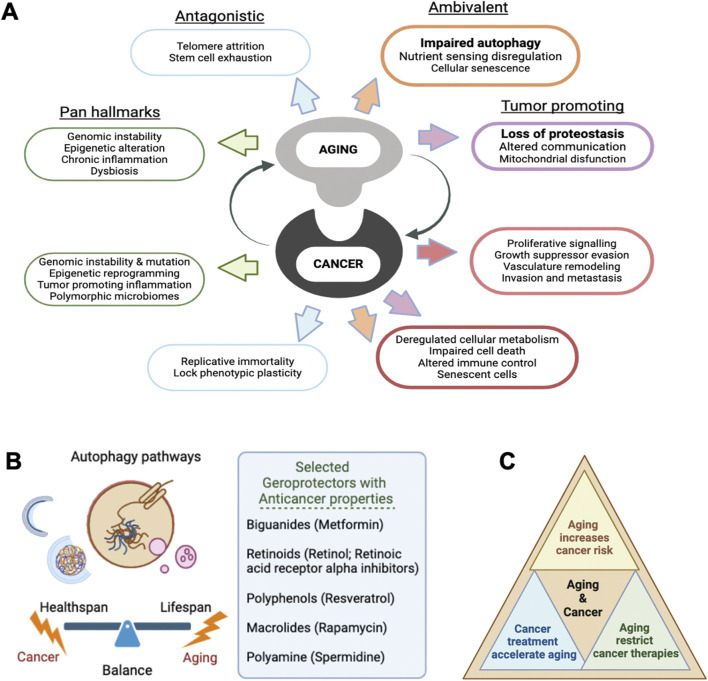
**(A)** Schematic illustration highlighting the close relationship between aging and cancer, summarizing key hallmarks of aging in four established groups alongside the related hallmarks of cancer. Both aging and cancer share multiple biological mechanisms that influence each other, including proteostasis and autophagy, highlighted on the right side of the scheme. **(B)** Illustration of the role of autophagy pathways in maintaining cellular balance, highlighting their impact on healthspan and lifespan, with implications for combating aging and cancer. **(C)** Age and frailty profoundly affect cancer risk, tumor biology. Factors such as treatment options, toxicity, tolerability, and effectiveness are heavily shaped by age, with frailty playing an even more significant role.

These **hallmarks in cancer** overlap with **aging’s hallmarks**, including genetic mutations, epigenetic changes, telomere shortening, cellular senescence, proteostasis disruption, and chronic inflammation (Hanahan, 2022), all contributing to cancer development and progression (Figure 1A).

## 4 Autophagy: a crucial link between aging and cancer

One key process that connects both aging and cancer is autophagy. Autophagy is an essential mechanism by which cells degrade and recycle cellular components, thus maintaining homeostasis and controlling a variety of essential cellular processes. This term encompasses three well-differentiated processes: macroautophagy (including selective types), microautophagy, and chaperone-mediated autophagy (CMA) (Schneider and Cuervo, 2014).

All three types of autophagy coexist in mammal cells and can compensate for each other when one is impaired. However, data from different studies suggest that the crosstalk between the different autophagic processes is complex and likely context-dependent (Kaushik et al., 2021). As we age, the efficiency of autophagy tends to decline, contributing to the aging process and increasing susceptibility to cancer. We explore here the complex role of autophagy as a bridge between aging and cancer, highlighting its impact on cellular homeostasis with aging-related decline and tumor development.

### 4.1 Autophagy’s influence on the aging process

Age-related decline in autophagy leads to expansion of the lysosomal compartment, an increase in the levels of certain lysosomal proteases -but a decline in their proteolytic activity, and accumulation of undegraded material within lysosomes.

#### 4.1.1 Macroautophagy and aging

There is a well-established link between macroautophagy and lifespan extension. Studies show that loss-of-function mutations in ATGs shorten lifespan, while increased macroautophagy, particularly under dietary restriction, extends it (Hansen et al., 2008). However, age-related macroautophagy decline varies by organ, and life-extending interventions are often organ-specific (Chang et al., 2017).

In mammals, aged rodents display a reduced autophagic flux (Del Roso et al., 2003; Donati et al., 2001; Terman, 1995), but interestingly, long-lived species and centenarians tend to maintain robust autophagic function even at advanced ages (Pérez et al., 2009; Raz et al., 2017). Age-related macroautophagy decline may stem from defective autophagosome-lysosome trafficking, hindering their fusion. Moreover, this autophagic dysfunction is characterized by downregulation of key autophagy effectors like Beclin-1, Atg5, Atg7 (Lipinski et al., 2010; Shibata et al., 2006), LC3 and Atg7 (Kaushik et al., 2012), and an increase in negative regulators like Rubicon (Nakamura et al., 2019). Altogether renders in the accumulation of defective mitochondria, increased oxidative stress, and neurodegeneration, contributing to age-related disorders.

It is also relevant to mention the selective processes, including mitophagy -used as a mechanism to repair mitochondrial DNA and proteins to maintain the mitochondrial network; its efficiency has been shown to decline with age (D'Arcy, 2024), as Parkin and PINK1 encompassing BNIP3/NIX, FUNDC1, and Bcl2-L-13 prompts the accumulation of dysfunctional mitochondria, which contributes to carcinogenesis (Denisenko et al., 2021).

Currently, research efforts are focused on strategies to upregulate autophagy genes or prevent the decline of autophagy, to extend lifespan and improve health span in mammalian models like mice.

#### 4.1.2 CMA in aging

Chaperone-mediated autophagy activity has been reported to decline with age in nearly all cell types and tissues in both rodents and humans (Kaushik et al., 2021; Schneider et al., 2015; Valdor et al., 2014; Zhang and Cuervo, 2008). This decline is primarily attributed to reduced LAMP2A levels in aging organisms (Kaushik et al., 2021). However, genetic restoration of LAMP2A in aged mice has been shown to effectively reduce proteotoxicity and preserve cellular function (Dong et al., 2021; Zhang and Cuervo, 2008), by maintaining protein quality control (Dong et al., 2021; Zhang and Cuervo, 2008; Bourdenx et al., 2021), and regulating specific cellular processes like glycolysis or endocytosis (Dong et al., 2021; Bourdenx et al., 2021).

Although inhibition of CMA and macroautophagy generates similar outcomes, both pathways are complementary and non-redundant, as they target distinct subsets of proteins and cellular components for degradation (Kaushik et al., 2021).

#### 4.1.3 Microautphagy and aging

Microautophagy has been less extensively studied compared to other forms of autophagy, so its role in the aging process remains largely unknown. However, evidence suggests that with age, there is an accumulation of carbonylated proteins and lipid peroxidation products in multivesicular bodies (MVBs), which would indicate a deterioration in endosomal microautophagy (eMI) (Cannizzo et al., 2012).

In summary, autophagy and aging are tightly interconnected, with autophagy playing a critical role in many hallmarks of aging, and preventing physiological and developmental problems like neurodegeneration, liver failure and cancer. Defective autophagy leads to accumulation of damaged proteins, causing mitochondrial dysfunction, oxidative stress and inflammation. In addition, autophagy regulates metabolism, prevents excessive apoptosis and modulates the immune system (Schneider et al., 2014; Tabibzadeh, 2023).

For a broader and extended review related the role of autophagy in aging-related disorders there has been recently published (Wu et al., 2024; Cassidy and Narita, 2022).

### 4.2 The impact of autophagy in cancer

Autophagy plays a dual and complex role in cancer, acting both as a tumor suppressor and as a promoter of tumor growth depending on the context. This role varies with the specific type of cancer, the stage of tumor development, and the tumor microenvironment.

#### 4.2.1 Autophagy as a tumor suppressor

Aging impairs the protective role of autophagy against cancer development. As a tumor suppressor, autophagy regulates key factors such as cell proliferation, genomic instability (Mathew et al., 2009), necrosis, inflammation (Tang et al., 2010), and oxidative stress (Tabibzadeh, 2023). Proteins such as Beclin-1, ATG5, ATG7, BNIP3, and BNIP3L, are essential in tumor suppression (Tabibzadeh, 2023; Yang Z. J. et al., 2011; Debnath et al., 2023). Additionally, accumulation of p62/SQSTM1, in autophagy-deficient cells increases. DNA damage and genomic instability, and its removal protects against carcinoma development (Mathew et al., 2009; Duran et al., 2008). Autophagy also limits necrosis and chronic inflammation reinforcing its tumor-suppressive role (Tang et al., 2010).

Moreover, CMA -a selective type of autophagy, is key in cancer, with increased activity in several tumors and cell lines. Blocking CMA reduces survival, tumorigenicity and tumor growth (Gomez-Sintes and Arias, 2021).

#### 4.2.2 Autophagy as a tumor growth promoter

Autophagy can act as a promoter of tumor growth under certain conditions. Aging promotes a proinflammatory environment, in which cancer cells often emerge. Once transformed, they may upregulate autophagy as an adaptative mechanism to overcome stressors such as accumulation of senescent cells and oxidative stress, supporting their growth and survival.

In established tumors, autophagy is essential for cancer cell survival, particularly by enabling cells to tolerate cytotoxic and metabolic stressors, such as hypoxia -via HIF-1α (Semenza, 2010), and nutrient deprivation. Tumor cells often have high metabolic demands, and autophagy allows them to recycle ATP and maintain crucial biosynthetic processes for growth and survival (Yang Z. J. et al., 2011).

Additionally, autophagy may support tumor recurrence and progression by promoting dormancy in cancer cells that survive chemotherapy and/or radiotherapy (Lu et al., 2008). Elevated basal levels of autophagy have been detected in different cancer cells, including pancreatic cancer and tumors with *H-ras* or *K-ras* mutations (Yang Z. J. et al., 2011). Inhibition of autophagy in those tumors leads to tumor regression and improve survival, suggesting that targeting autophagy could improve the efficiency of cancer therapies (Yang S. et al., 2011; Guo et al., 2011).

Following malignant transformation, cancer cells frequently upregulate CMA, contributing to tumor growth and proliferation by sustaining the Warburg effect, and providing protection against cytotoxic and chemical agents, modulating immune cell populations in the tumor microenvironment, and degrading tumor suppressors, pro-apoptotic and anti-proliferative factors (Gomez-Sintes and Arias, 2021).

Other selective types as mitophagy also show a pro-tumorigenic role that can vary depending on the cancer type and stage (Song et al., 2022). In glioblastoma and triple-negative breast cancer, for example, it contributes to enhance tumor cell proliferation and metastasis (Dong and Zhang, 2024).

While we have growing understanding of how autophagy promotes tumor suppression, survival and progression, further research is necessary across different tumor types to fully elucidate how this process can either inhibit or promote cancer development. This knowledge is crucial for designing targeted therapies tailored to specific cancers.

### 4.3 Non-canonical autophagy processes

Research on alternative mechanisms of autophagy is rapidly expanding. These mechanisms, collectively referred to as non-canonical autophagy (NCA), have been identified under specific cellular conditions and hold particular relevance in cancer (Debnath et al., 2023). Unsurprisingly, our understanding of how tumors and associated cells adapt NCA pathways to support tumor growth and progression, especially in the context of aging, is also advancing. Among these alternative pathways, notable examples include: (I) LC3-associated processes: The finding of phagocytic vesicles decorated with LC3 revealed a non-classical function of ATG proteins beyond autophagosome formation (Sanjuan et al., 2007). Posterior studies further expanded this process of LC3-associated phagocytosis (LAP) and identified LAP-like LC3 conjugation on endosomes (Jacquin et al., 2017), LC3-associated endocytosis (LANDO) (Heckmann et al., 2019) and LDELS [(LC3)-dependent extracellular vesicle (EV) loading and secretion] (Leidal et al., 2020). (II) Autophagic membranes as signaling platforms: autophagy-deficient mice show reduced oncogenic signaling through well-known pathways as the AKT–PI3K and MAPK-ERK (Karsli-Uzunbas et al., 2014; Fraser et al., 2017; Martinez-Lopez et al., 2013). This may be due to the tumor-promoting roles of autophagy, or interactions between autophagy components and growth factor signaling pathways. (III) Autophagy-Independent Functions of ATG Proteins in Tumorigenesis: ATG proteins can exert non-autophagy-related functions that significantly influence immune response, vesicular trafficking, cell death and tumorigenesis (Galluzzi and Green, 2019), a critical consideration when targeting autophagy in cancer therapy.

Not all cargoes from NCA undergo lysosomal degradation. Due to the diverse outcomes of cargo processing, NCA can function either as a degradative or a secretory pathway. Recent studies illustrating the importance of autophagy in the host stroma have coincided with a growing appreciation in the field that autophagy controls extracellular secretion (Deretic et al., 2012; Ponpuak et al., 2015).

### 4.4 Therapeutic advances

Given the dual role of autophagy in both aging and cancer, researchers are now exploring therapies that modulate autophagy in a more detailed strategy. In healthy individuals, different studies have shown that restoring autophagy levels in aged organisms can reduce tumor formation. On the other hand, for aging population with cancer, maintaining a delicate balance between activating autophagy to support healthy aging and inhibiting it to prevent tumor growth in the tissues affected is a central challenge. Precision medicine approaches that tailor autophagy modulation based on an individual’s age, cancer type, and overall health status are emerging as a promising area of therapeutic innovation.

When focusing on aged cancer patients, different strategies have been explored, summarized in Table 1. Among them, several compounds considered geroprotectants, such as biguanides (metformin) (Zajda et al., 2020), retinoids (Hałubiec et al., 2021), polyphenols (resveratrol) (Raj et al., 2021; Mundo Rivera et al., 2024), rapamycin (Juricic et al., 2022), and polyamines (spermidine) (Hofer et al., 2022; Zimmermann et al., 2023), are currently being studied for their ability to activate autophagy, making them exciting compounds for their potential use in cancer treatment (Figure 1B).

**TABLE 1:** Autophagy modulators for cancer therapy.

Therapy	Type	Target	Cancer type	Refs
Macroautophagy				
SBI-0206965	Inhibitor	ULK1/2	Non-small cell lung cancer	Egan et al. (2015), Lee et al. (2023), and Lim and Murthy (2020)
			AML	
MRT68921	Inhibitor	ULK1/2	Lung	Lee et al. (2023), Lim and Murthy (2020), Petherick et al. (2015), and Martin et al. (2018)
			Gastric	
ULK-101	Inhibitor	ULK1/2	Renal	Martin et al. (2018), and Hassan et al. (2024)
			Non-small cell lung cancer	
3-MA	Inhibitor	VPS34/PIK3C3	Uterine sarcoma	Petiot et al. (2000)
SAR405	Inhibitor	VPS34/PIK3C3	Renal cell carcinoma	Ronan et al. (2014), and Pasquier (2015)
SB02024	Inhibitor	VPS34/PIK3C3	Breast cancer	Noman et al. (2020)
VPS34-IN1	Inhibitor	VPS34/PIK3C3	AML	Bago et al. (2014)
NSC185058	Inhibitor	ATG4B	Glioblastoma	Akin et al. (2014)
			Osteosarcoma	
UAMC-2526	Inhibitor	ATG4B	Colorectal cancer	Kurdi et al. (2017)
S130	Inhibitor	ATG4B	Colorectal cancer	Fu et al. (2019)
Tioconazole	Inhibitor	ATG4B	Breast cancer	Liu et al. (2018), and El-Gowily et al. (2021)
Cloroquine*	Inhibitor	Lysosome	Hepatocellular carcinoma	Lee et al. (2023), Lim and Murthy (2020), and Levy et al. (2017)
			Breast	
			Glioblastoma	
Hydroxychloroquine (HCQ)*	Inhibitor	Lysosome	Hepatocellular carcinoma	Lee et al. (2023), Lim and Murthy (2020), and Amaravadi et al. (2019)
			Breast	
			Glioblastoma	
			Pancreatic cancer	
			Prostate cancer	
ROC-325	Inhibitor	Lysosome	AML	Nawrocki et al. (2019)
DC661	Inhibitor	Lysosome	Hepatocellular carcinoma	Rebecca et al. (2019)
SF1126	Inhibitor	PI3 Kinase, mTOR	Neuroblastoma	Hassan et al. (2024)
Temsirolimus	Inhibitor	S6 Kinase, mTOR	Ovarian carcinoma	Hassan et al. (2024)
			Advance endometrial carcinoma Advanced liver cancer	
MK-2206	Inhibitor	AKT	Colorectal cancer	Hassan et al. (2024)
Bortezomib	Inhibitor	Proteasome	Lymphoma	Hassan et al. (2024)
Everolimus	Inhibitor	mTOR	Advanced HCC	Hassan et al. (2024)
			Bladder	
			Metastatic transitional cell carcinoma	
Sorafenib	Inhibitor	Tyrosine kinase	Advanced HCC	Hassan et al. (2024)
Pevonedistat	Activator	NEDD-9	AML	Mohsen et al. (2022)
			Melanoma	
			MDS	
SAHA	Activator	mTOR	Cutaneous T-cell lymphoma	Gammoh et al. (2012)
			Glioblastoma	
Mitophagy				
Atovaquone	Inhibitor	Complex III, OXPHOS	Non-small cell lung cancer (pancreatic, breast and brain cancer cell lines)	Villa-Ruano et al. (2023)
		Glycolysis		
Honokiol	Inhibitor	Complex I	Early-Stage Resectable Non-Small Cell Lung Cancer	Zhang et al. (2022)
		STAT3 phosphorylation		
Lonidamine	Inhibitor	Complexes I/II	Benign Prostatic Hyperplasia	Zhang et al. (2022)
		AKT/mTOR/p70S6K signaling		
Geroprotectants				
Metformin		AMPK/mTOR	Colorectal	Markowska et al. (2022), Phillips et al. (2023), and Zhang et al. (2023)
			Lung	
			Pancreatic cancer	
			Gynecological cancers	
Resveratrol	Activator	AMPK/mTOR	Breast	Behroozaghdam et al. (2022), Roshani et al. (2022), and Xin et al. (2023)
			Gastrointestinal	
			Lung	
Rapamycin	Activator	mTOR	*Preventive treatment or as coadjuvant*	Blagosklonny (2023), and Kemp Bohan et al. (2021)
Spermidine	Activator	MAP1S	Hepatocellular carcinoma	Prasher et al. (2023), Pietrocola et al. (2019), Xu et al. (2016), and Chen et al. (2018)
			Renal cell carcinoma	
			Prostate adenocarcinoma	
			Cervical cancer	

Most in the list are in pre-clinical stages and have demonstrated efficacy in decreasing tumor growth and proliferation when used in monotherapy. Some are already FDA-approved for treatment of other diseases. Currently, several studies are evaluating the use of these compounds in combination with chemotherapy or other drugs (U.S. Department of Health and Human Services, 2024).

* Both CQ and HCQ are repurposed drugs for cancer therapy and currently several clinical trials are using them in combination with other therapies.

Of particular note is the concept of *“the autophagic switch”*, where autophagy shifts from cytoprotective to cytotoxic, opening the possibility of more effective treatments (Frentzel et al., 2017). The complexity and specificity of autophagy pathways emphasizes the need for further research on these cellular mechanisms.

In metastatic cancer cells, autophagy often becomes upregulated, helping these cells survive under the harsh conditions they encounter during invasion and colonization, such as nutrient deprivation, oxidative stress, and immune surveillance. This ability to use autophagy to adapt and thrive in new environments makes it a key driver of metastasis. However, autophagy also has a dual role in metastasis that is stage specific, and in later stages, it can suppress metastatic colonization (Marsh et al., 2021). Therapeutically, targeting autophagy has become a promising approach in the treatment of metastatic cancers. Inhibitors are currently being tested in combination with other cancer therapies to limit not only the survival but also the dissemination of metastatic cells.

## 5 Concluding remarks

The relationship between aging and cancer is thus highly complex and ambivalent, with certain mechanisms, like autophagy, that can both hinder and fuel cancer progression. Understanding the interactions would lead to better therapeutic strategies that balance cancer treatment with managing the effects of aging.

Future research should explore how geroprotective measures, including intervention of autophagy processes, might be integrated with cancer therapies without compromising their efficacy. The collaboration between oncology and geriatric medicine is essential, particularly as population is reaching older ages, cancer is most often diagnosed in older adults, and oncological treatments accelerate aging (Figure 1C). Clinical trials exploring biological aging markers could also provide more personalized treatment strategies by considering a patient’s biological rather than chronological age. This could pave the way for safer, more effective treatment approaches that account for the interplay between aging and cancer.
